# The flow-metabolism ratio might predict treatment response and survival in patients with locally advanced esophageal squamous cell carcinoma

**DOI:** 10.1186/s13550-020-00647-9

**Published:** 2020-05-29

**Authors:** Kewei Zhao, Chunsheng Wang, Qingfeng Mao, Dongping Shang, Yong Huang, Li Ma, Jinming Yu, Minghuan Li

**Affiliations:** 1 School of Medicine, Shandong University, Wenhua West Road 44, Jinan, 250012 Shandong Province China; 2 Department of Radiation Oncology, Shandong Cancer Hospital and Institute, Shandong First Medical University and Shandong Academy of Medical Sciences, Jiyan Road 440, Jinan, 250117 Shandong Province China; 3 Department of Radiation Oncology, Qingdao University Medical College Affiliated Yantai Yuhuangding Hospital, Yantai, China; 4 Department of Radiation Oncology, Jiangxi Cancer Hospital Affiliated to Nanchang University, Nanchang, China; 5 Department of Radiology, Shandong Cancer Hospital and Institute, Shandong First Medical University and Shandong Academy of Medical Sciences, Jinan, China; 6 Department of Nuclear Medicine, Shandong Cancer Hospital and Institute, Shandong First Medical University and Shandong Academy of Medical Sciences, Jinan, China

**Keywords:** Locally advanced, Esophageal squamous cell cancer, Definitive chemoradiotherapy, Flow-metabolism ratio, Perfusion CT, ^18^F-FDG PET/CT

## Abstract

**Background:**

Perfusion CT can offer functional information about tumor angiogenesis, and ^18^F-FDG PET/CT quantifies the glucose metabolic activity of tumors. This prospective study aims to investigate the value of biologically relevant imaging biomarkers for predicting treatment response and survival outcomes in patients with locally advanced esophageal squamous cell cancer (LA ESCC).

**Methods:**

Twenty-seven patients with pathologically proven ESCC were included. All patients had undergone perfusion CT and ^18^F-FDG PET/CT using separate imaging systems before receiving definitive chemoradiotherapy (dCRT). The perfusion parameters included blood flow (BF), blood volume (BV), and time to peak (TTP), and the metabolic parameters included maximum standardized uptake value (SUVmax), metabolic tumor volume (MTV), and total lesion glycolysis (TLG). The flow-metabolism ratio (FMR) was defined as BF divided by SUVmax. Statistical methods used included Spearman’s rank correlation, Mann–Whitney *U* test or two-sample *t* test, receiver operating characteristic (ROC) curve analysis, the Kaplan–Meier method, and Cox proportional hazards models.

**Results:**

The median overall survival (OS) and progression-free survival (PFS) were 18 and 11.6 months, respectively. FMR was significantly positively correlated with BF (*r* = 0.886, *p* < 0.001) and negatively correlated with SUVmax (*r* = − 0.547, *p* = 0.003) and TTP (*r* = − 0.462, *p* = 0.015) in the tumors. However, there was no significant correlation between perfusion and PET parameters. After dCRT, 14 patients (51.9%) were identified as responders, and another 13 were nonresponders. The BF and FMR of the responders were significantly higher than those of the nonresponders (42.05 ± 16.47 vs 27.48 ± 8.55, *p* = 0.007; 3.18 ± 1.15 vs 1.84 ± 0.65, *p* = 0.001). The ROC curves indicated that the FMR [area under the curve (AUC) = 0.846] was a better biomarker for predicting treatment response than BF (AUC = 0.802). Univariable Cox analysis revealed that of all imaging parameters, only the FMR was significantly correlated with overall survival (OS) (*p* = 0.015) and progression-free survival (PFS) (*p* = 0.017). Specifically, patients with a lower FMR had poorer survival. Multivariable analysis showed that after adjusting for age, clinical staging, and treatment response, the FMR remained an independent predictor of OS (*p* = 0.026) and PFS (*p* = 0.014).

**Conclusions:**

The flow-metabolism mismatch demonstrated by a low FMR shows good potential in predicting chemoradiotherapy sensitivity and prognosis in ESCC.

## Background

Esophageal squamous cell carcinoma (ESCC) is the most common pathological subtype of esophageal cancer (EC) in Asian countries, including China, accounting for approximately 90% of cases [1]. Definitive chemoradiotherapy (dCRT) is the standard therapy for patients with locally advanced ESCC (LA ESCC) who have unresectable tumors, advanced age, or severe heart and lung disease or who refuse surgery [2]. For these patients, to choose the appropriate type and intensity of treatment for individualization, it is important to find reliable biomarkers to predict prognosis and chemoradiotherapy sensitivity. The tumor lymph node metastasis (TNM) staging system is considered to be the most powerful prognostic factor [3], but it is only an anatomical description that cannot reflect tumor aggressiveness, and even for diseases at the same stage, the prognosis may be very different. Therefore, the application of “functional” molecular imaging is gradually emerging because of its ability to detect the physiological and cellular processes and reflect the biological characteristics of tumors, thus surpassing conventional morphological imaging.

Uncontrolled, disordered, and sustained angiogenesis is a hallmark of cancer [4]. Dynamic contrast-enhanced (DCE) perfusion computed tomography (CT), referred to as perfusion CT, is a functional imaging technique that can offer both qualitative and quantitative information about tumor angiogenesis [5, 6]. Although angiogenesis plays an essential role in tumor growth, it also provides a powerful condition for the delivery of chemotherapy drugs and improvement of radiotherapy resistance caused by hypoxia [5]. Many studies have confirmed the value of perfusion biomarkers in monitoring the therapeutic response and long-term survival of solid tumors [7–11]. However, in ESCC, the data are limited [10, 11].

Abnormal glucose metabolism is another hallmark of cancer and is considered to be an important marker of tumor biological behavior [4]. ^18^F-fluoroglucose glucose positron emission tomography (^18^F-FDG PET/CT) is another molecular imaging technology that can reflect the glucose metabolism rate of tumors by quantifying FDG uptake. To date, it has been widely applied in ESCC patients for diagnosis, staging, response evaluation, and survival prediction [12]. Although many studies have investigated the predictive value of FDG-derived metabolic biomarkers for tumor response to chemoradiation and prognosis, the conclusions are inconsistent, suggesting that FDG uptake alone is not sufficient for predicting outcomes [13–15].

In recent years, multiparametric imaging approaches have been increasingly applied in studies of various tumors, especially the combination of perfusion imaging and ^18^F-FDG PET/CT, providing novel insights into delineating tumor characteristics and aggressiveness [16–22]. In pancreatic cancer and breast cancer, investigators found that the relationship between blood flow (BF) and glucose metabolism is a potential indicator of the biological status of tumors, especially the low-BF and high-metabolism phenotype (i.e., showing a mismatch between flow and metabolism), which has been shown to be associated with clinically more aggressive tumors, predicting poorer patient outcomes [16, 17]. However, in EC, although there have been studies on tumor perfusion [10, 11] and glucose metabolism [12–15], no studies have evaluated these two factors simultaneously.

Hence, this prospective study was conducted in LA ESCC to assess tumor vascularization and metabolic activity in parallel using perfusion CT and FDG PET/CT. Our aims included (1) the investigation of the relationships between tumor perfusion and glucose consumption in vivo and (2) the identification of valuable imaging biomarkers to predict tumor response to chemoradiotherapy and survival outcomes.

## Methods

### Patient selection

Between January 2015 and September 2017, thirty-one patients newly diagnosed with ESCC and planning to undergo dCRT in Shandong cancer hospital and institute (Jinan, China) were enrolled, and all met the following inclusion criteria: 18–75 years old; pathologically proven diagnosis of ESCC; no prior treatment; Eastern Cooperative Oncology Group performance status (ECOG PS) 0–2; clinical stage IIb to III based on the 7th edition of the AJCC TNM classification; adequate hematological, liver and kidney function; and no history of other malignancies. The main exclusion criteria were pregnancy and a known allergy to intravenous contrast agents. All 31 patients underwent integrated perfusion CT/^18^F-FDG PET before treatment to assess tumor perfusion [BF, blood volume (BV), time to peak (TTP)] and metabolism parameters [standardized uptake value (SUV), metabolic tumor volume (MTV), and total lesion glycolysis (TLG)]. This prospective study was approved by the Ethical Committee of Shandong Cancer Hospital and Institute. Informed consent was obtained from all participants prior to their inclusion in the study.

### Perfusion CT protocol and image analysis

The perfusion imaging was performed on a 16-slice Philip Brilliance big bore CT scanner (Philip Brilliance big bore CT; Philips Medical Systems; the Netherlands). The CT protocol includes two procedures. The first is unenhanced scanning of the neck/chest/upper abdomen to determine the tumor location; eight adjacent sections containing the maximum diameter of the lesion center were selected for cine imaging. The parameters used were as follows: slice thickness, 3 mm; speed, 30 mm/sec; pitch, 0.94; 120 kV; 120 mA; scan field of view, 30 cm; and matrix, 512 mm × 512 mm. The second step is a dynamic enhanced perfusion scan. In this step, the patients were first intravenously injected with 50 mL of a non-ionized iodinated contrast agent (300 mg/mL) at a rate of 5 mL/s through an 18-gauge cannula in the median cubital vein. After a 6-second delay, a dynamic scan (120 kV, 120 mAs, 30-cm scan field of view, 512 × 512 matrix) of the predetermined levels of interest (eight adjacent sections with 3-mm reconstructed section thickness and a total of 24 mm) was started and lasted for 40 s (0.5 s scan time and 1.5 s interval).

**Fig. 1:**
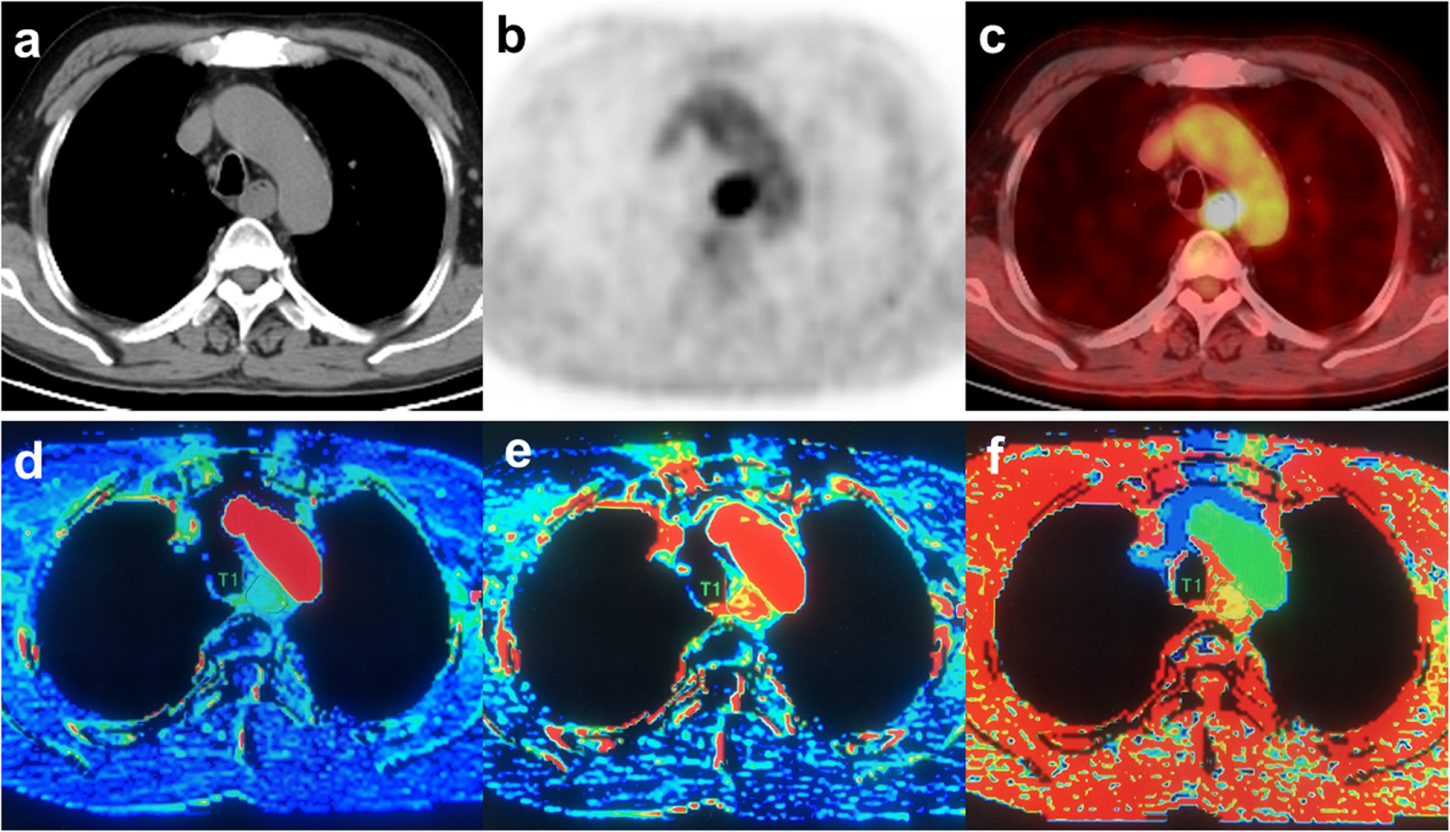
A 75-year-old man with a squamous cell carcinoma in the upper thoracic portion ofesophagus. **a** CT image. **b** PET image. **c** Fused axial PET/CT image (SUVmax, 14.57; MTV, 16.32 mL; TLG, 92.91). **d** BF (47.3 mL/min/100 g). **e** BV (20.2 mL/100 g). **f** TTP (16.7 s)

All CT images obtained were transferred to a workstation (Extended Brilliance Workspace V3.5.0, Philips Healthcare) and were analyzed using commercially available software (Brilliance perfusion 2.1.1, Perfusion CT; Philips Healthcare) based on the maximum slope method. We selected the descending aorta as the inflow artery and defined the arterial input as that through circular regions of interest (ROIs). Then, time-density curve (TDC) and functional perfusion maps were automatically generated (Fig. 1). If the lesion was located at the superior level of the aortic arch, the brachial trunk or the left common carotid artery was selected as the inflow artery. Two experienced radiologists manually delineated the ROI of the lesion in such a way that it contained as many solid tumor portions as possible while avoiding the esophageal cavity and paraesophageal fat, cysts, and necrotic areas, and had an area greater than 40 mm^2^. Finally, the average values of BF (in mL/100 g/min), BV (in mL/100 g), and TTP (in sec) were recorded as perfusion parameters for further analysis.

### PET/CT scanning and image analysis

^18^F-FDG PET/CT scanning was performed on an advanced PET/CT scanner (Discovery LS, GE Healthcare). All patients were required to fast, rest quietly for more than 6 h before the scan, and ensure that their blood glucose concentrations were less than 6 mmol/L. Then, they were injected with 5.50–7.40 MBq/kg of ^18^F-FDG intravenously. After 60 min, whole-body PET and CT scans were performed from the top of the skull to the proximal thigh using the following scanning parameters: 140 kV, 80 mA, 6:1 pitch, 4.25-mm section thickness, 50-cm field of view, and 512 × 512 matrix. Patients were advised to relax and maintain normal shallow breathing during image acquisition. After CT-derived attenuation correction, the PET images were reconstructed by the ordered subset expectation maximization algorithm. The PET, CT, and fused images were transmitted to a Xeleris workstation (GE Healthcare) for transverse, coronal, and sagittal display.

All images were independently assessed by two experienced nuclear medicine radiologists, and if there were inconsistencies, they were discussed until a consensus was reached. The values of SUV, MTV, and TLG were obtained through a vendor-provided automated contouring program. SUVmax was selected as the parameter to quantify FDG uptake, defined as the maximum voxel value of the SUV within the ROI. MTV represents the volume of tumor tissue with higher metabolic activity than the surrounding normal tissue, which is measured by fixed threshold method (setting the threshold value of SUV as 2.5) in this study. TLG is the product of MTV and SUVmean, representing the combined volumetric and metabolic tumor burden. The flow-metabolism ratio (FMR) is defined as BF divided by SUVmax.

### Treatment protocol

All patients were treated with dCRT in a concurrent pattern. The radiotherapeutic technique used by all patients was the three-dimensional conformal radiation therapy or intensity-modulated radiation therapy by high-energy linear accelerators (6 MV). The gross tumor volume (GTV) included the primary tumor (GTVp) and metastatic lymph nodes (GTVnd), as shown by CT, PET/CT, esophagography, endoscopy, or endoscopic ultrasonography. The criteria for metastatic lymph nodes are those with a short diameter exceeding 1.0 cm or a long diameter exceeding 1.5 cm or those with a high ^18^F-FDG uptake on PET images (SUV ≥ 2.5). The clinical target volume (CTV) included the primary tumor plus a 3-cm craniocaudal margin and a 1.0-cm margin in other directions as well as the metastatic lymph nodes plus a 1.0-cm margin. Planning target volume (PTV) was generated by 5-mm outward expansion of CTV in all directions. Considering that the radiation dose of 50.4 Gy may not sufficient for local control, all patients in our study received a total dose of 60–64 Gy with 30–32 fractions, which is a popular radiation dose in China. Chemotherapy started on day 1 of the initial radiation. The regimens consisted of cisplatin (75 mg/m^2^ on day 1) plus 5-fluorouracil (750 mg/m^2^ on days 1-3) or cisplatin (25 mg/m^2^ on days 1–3) plus docetaxel (60 mg/m^2^ on day 1), which were repeated every 21 days. Patients underwent 2 cycles of chemotherapy during radiation. After radiation, they received another 2 to 4 cycles of additional chemotherapy.

### Assessment of treatment response and follow-up

Four weeks after treatment completion, based on comparison of the chest enhanced CT, esophagography before and after treatment, combined with the changes in patients’ clinical symptoms (such as dysphagia), the tumor response to dCRT was evaluated. According to the Revised Response Evaluation Criteria in Solid Tumors (RECIST 1.1) criteria, the responses were divided into 4 categories: complete response (CR, disappearance of all evaluable disease), partial response (PR, a decrease of at least 30% in the sum of maximum diameter of target lesions), progressive disease (PD, an increase of 20% or over in the sum of maximum diameter of target lesions or the appearance of new lesions), and stable disease (SD, a tumor response that did not fulfill the PR criteria but exceeded the PD criteria); patients who achieved CR and PR were considered responders, while those who were classified as PD and SD were identified as nonresponders. If the above examinations cannot determine the patient’s treatment response, the patient will be further scheduled for a second PET scan or biopsy to confirm. For example, using PET to determine the nature of newly emerging lesions during treatment (inflammatory or metastatic?), and use endoscope and biopsy to further determine the CR identified in imaging examination.

Follow-up was performed every 3 months in the first year after treatment, every 6 months in the second year, and then every year thereafter until death or the last follow-up date. During the follow-up, arrange the necessary examinations for the patient according to their condition, including tumor markers, barium meal, contrast-enhanced CT, PET/CT, endoscope, and biopsy. Overall survival (OS) was defined as the time interval between the start date of dCRT and the date of death of any cause or the date of the last follow-up. Progression-free survival (PFS) was defined as the time interval between the start date of dCRT and the date of diagnosis of cancer progression (including local recurrence and distant metastasis) or the date of death for any reason. Follow-up methods include appointments for outpatient visits and telephone consultations. The last follow-up date was September 18, 2019.

### Statistical analysis

All statistical analyses were performed using SPSS version 22.0 and MedCalc 18.2.1. All tests were 2-sided, and *p* < 0.05 was considered statistically significant. Quantitative parameters are expressed as the means ± standard deviations. Spearman’s rank correlation coefficient was used to assess the correlation between perfusion and metabolism parameters. The Mann–Whitney *U* test or two-sample *t* tests was used to compare these parameters between the responders and nonresponders. Receiver operating characteristic (ROC) curves were conducted to compare the ability of different factors to identify the responders. The Kaplan–Meier method was used to construct survival curves, and log–rank tests were used to compare the differences. Variables with *p* values less than 0.1 in univariable analysis were included in multivariable Cox proportional hazards model analysis.

## Results

### Patient characteristics

One patient was diagnosed with a second primary lung cancer during treatment, and three patients discontinued dCRT and switched to surgery. So, they were excluded from analysis. Ultimately, the data from 27 patients (20 males, 7 females; median age, 65 years; range, 50–75 years) were included in the analysis. All patients were in clinical TNM stage IIb to III (T3-4N0-2M0). All patients underwent baseline perfusion CT and ^18^F-FDG PET with a median interval of 7 days (range 3–10 days). The patient’s baseline characteristics are listed in Table 1.

**Table 1:** Clinicopathological characteristics of 27 patients with esophageal squamous carcinoma

Characteristics	No. of patients
Age (< 65 years/≥ 65 years)	14/13
Gender (male/female)	20/7
Location (upper/middle/lower)	9/12/6
Length (< 5 cm/≥ 5 cm)	14/13
Clinical stage (IIb/III)	8/19
Response (responder/non-responder)	14/13

### Correlations between perfusion and metabolic parameters

According to the Spearman’s correlation analysis, FMR was significantly positively correlated with BF (*r* = 0.886, *p* < 0.001) and negatively correlated with SUVmax (*r* = − 0.547, *p* = 0.003) and TTP (*r* = − 0.462, *p* = 0.015) in the tumors. However, there was no significant correlation between the perfusion parameters (BF, BV, and TTP) and the PET-derived parameters (SUVmax, MTV, and TLG) (all |*r*| < 0.2 and all *p* > 0.05) (Table 2).

**Table 2:** Correlations between perfusion and metabolic parameters

Variables	SUVmax	MTV	TLG	FMR
BF (mL/100 g/min)	r = − 0.142 p = 0.479	r = 0.063 p = 0.755	r = 0.037 p = 0.854	r = 0.886 p < 0.001
BV (mL/100 g)	r = − 0.022 p = 0.912	r = − 0.114 p = 0.570	r = − 0.066 p = 0.745	r = 0.226 p = 0.257
TTP (s)	r = 0.072 p = 0.721	r = − 0.091 p = 0.651	r = − 0.065 p = 0.747	r = − 0.462 p = 0.015
FMR	r = − 0.547 p = 0.003	r = − 0.075 p = 0.710	r = − 0.155 p = 0.440	NA

*BF* blood flow, *BV* blood volume, *TTP* time to peak, *SUVmax* maximum standardized uptake value, *MTV* metabolic tumor volume, *TLG* total lesion glycolysis, *FMR* flow-metabolism ratio

### Correlations between perfusion/metabolic parameters and response

After dCRT, 14 (51.9%) patients were classified as responders, including 4 CR and 10 PR, and the other half were identified as nonresponders (all 13 patients were SD). The baseline perfusion and metabolic parameters are listed in Table 3. BF was significantly higher in the responders than in the nonresponders (42.05 ± 16.47 vs 27.48 ± 8.55, *p* = 0.007, Table 3). In addition, the FMR was also significantly higher in the responders than in the nonresponders (3.18 ± 1.15 vs 1.84 ± 0.65, *p* = 0.001, Table 3). Other parameters, such as BV, TTP, SUV, MTV, and TLG, were not significantly different between the responders and the nonresponders.

**Table 3:** Perfusion and metabolic parameters between responders and non-responders

Variables	All patients	Responders	Non-responders	***p*** value
**Perfusion parameters**				
BF (mL/100 g/min)	34.49 ± 14.72	42.05 ± 16.47	27.48 ± 8.55	0.007
BV (mL/100 g)	23.19 ± 9.90	24.31 ± 11.57	22.16 ± 8.37	0.720
TTP (s)	37.33 ± 17.38	34.44 ± 18.22	40.02 ± 16.78	0.416
**Metabolic parameters**				
SUVmax	14.40 ± 2.86	13.36 ± 2.54	15.36 ± 2.88	0.068
MTV	29.76 ± 24.49	27.09 ± 28.51	32.22 ± 20.88	0.375
TLG	177.46 ± 160.63	155.49 ± 176.32	197.85 ± 148.24	0.325
**Flow-metabolism ratio**				
FMR	2.48 ± 1.13	3.18 ± 1.15	1.84 ± 0.65	0.001

*BF* blood flow, *BV* blood volume, *TTP* time to peak, *SUVmax* maximum standardized uptake value, *MTV* metabolic tumor volume, *TLG* total lesion glycolysis, *FMR* flow-metabolism ratio

**Fig. 2:**
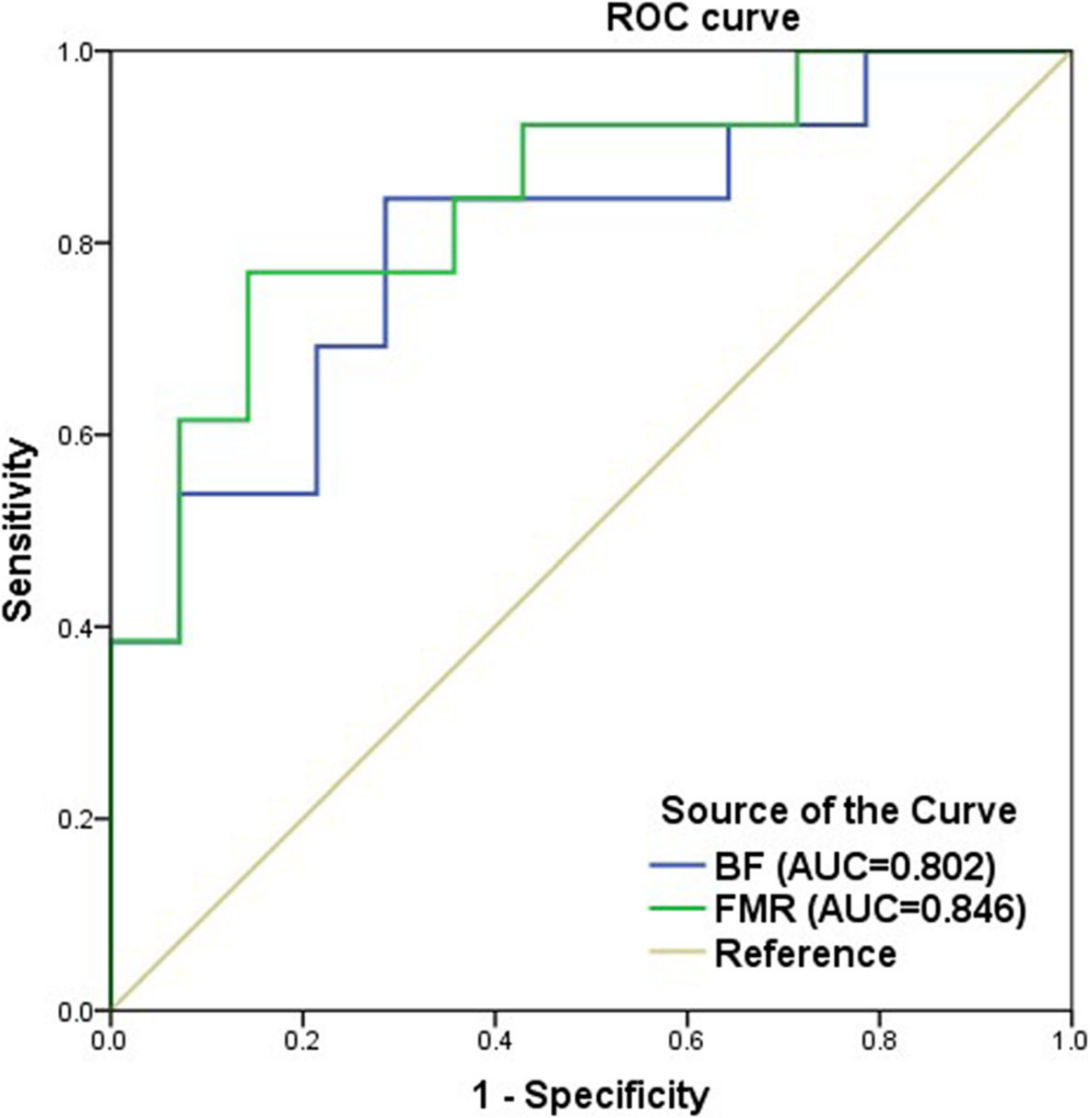
The AUCs of FMR and BF were 0.846 (95% CI 0.656-0.955; *p* = 0.002) and 0.802 (95% CI 0.605-0.929; *p* = 0.008). Comparison of ROC curves suggested that the FMR was a better biomarker for predicting treatment response than BF

ROC curves were constructed to assess the diagnostic accuracy of BF and FMR in identifying the responders. As shown in Fig. 2, the area under the ROC curve (AUC) for the FMR (AUC 0.846; 95% CI 0.656–0.955; *p* = 0.002) was higher than that for BF (AUC 0.802; 95% CI 0.605–0.929; *p* = 0.008), suggesting that the FMR was better for predicting response. However, the differences between AUC of BF and FMR were not statistically significant (ΔAUC = 0.044, *p* = 0.442; tested by MedCalc 18.2.1). This may be due to the small sample size that does not allow for sufficient statistical power.

ROC analysis revealed that the optimal thresholds of the FMR and BF for predicting treatment response were 2.38 (sensitivity 76.9%, specificity 85.7%) and 27.8 mL/100 g/min (sensitivity 84.6%, specificity 71.4%), respectively. The objective response rate (ORR) was significantly higher in patients with high BF than in those with low BF (71.4% vs 23.1%, *p* = 0.012). Similarly, there were also significant differences in the ORR between the high-FMR group and the low-FMR group (71.4% vs 23.1%, *p* = 0.012).

### Correlations between perfusion/metabolic parameters and survival

**Fig. 3:**
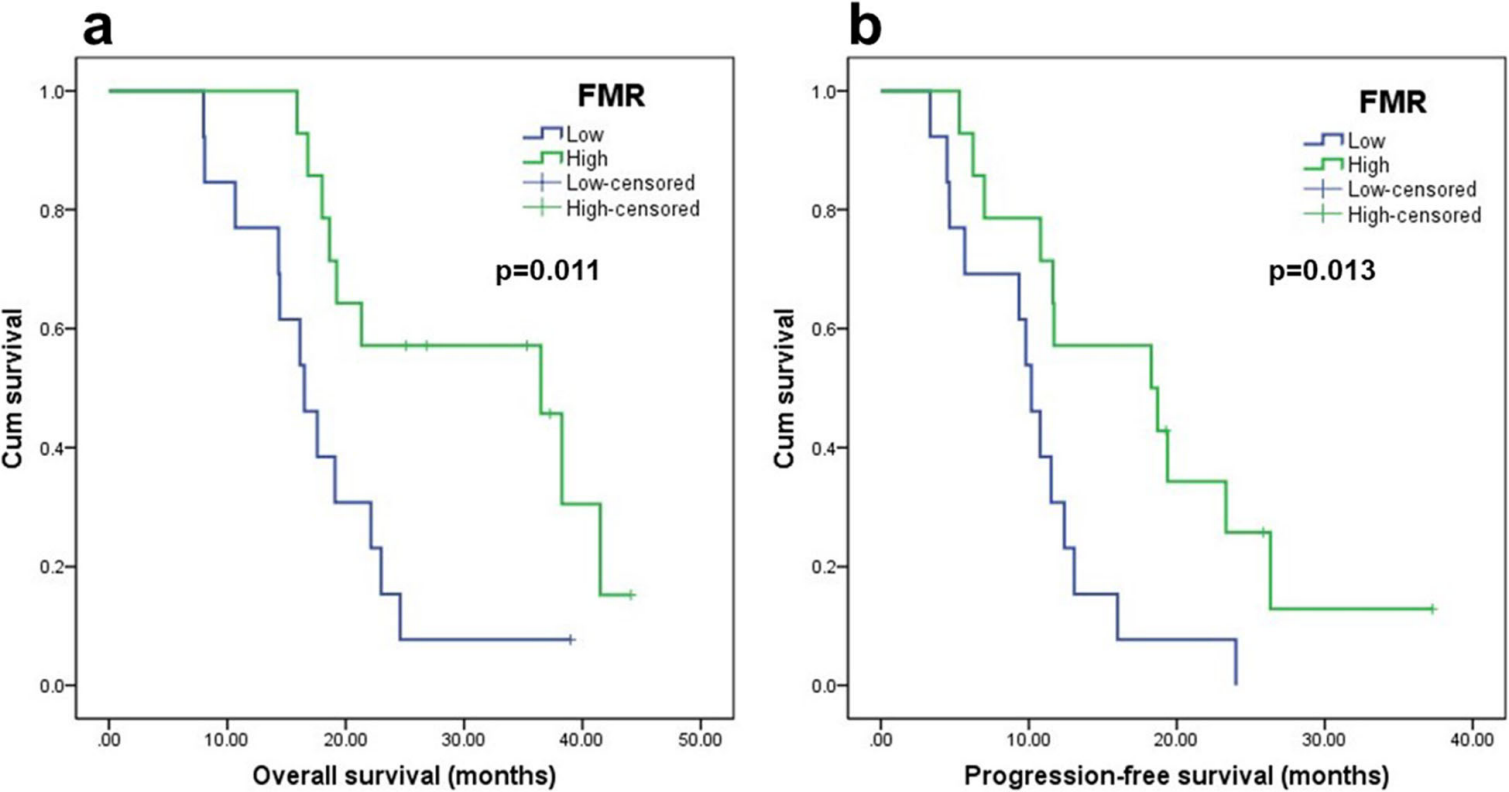
Kaplan–Meier survival curves showed that patients with low FMR have shorter **a** overall survival and **b** progression-free survival than those with high values

The median follow-up period of the all patients was 19.2 months (8–44.1 months) with a minimum of 24 months for living patients. The median OS was 18 months (range, 8–41.5 months), and the median PFS was 11.6 months (range, 3.3–26.3 months). The patients were dichotomized into 2 groups according to the median values of each parameter. The Kaplan–Meier curves showed that the patients with a high FMR exhibited a longer OS (*p* = 0.011, Fig. 3a) and PFS (*p* = 0.013, Fig. 3b) than those with a low FMR. However, there was no significant correlation between survival and other parameters.

Table 4 shows the results of the univariable analysis. The multivariable Cox model of OS and PFS included age, clinical stage, treatment response, and FMR. The results revealed that FMR was independently related to OS (HR 2.914; 95% CI 1.138–7.462; *p* = 0.026) and PFS (HR 3.009; 95% CI 1.252–7.227; *p* = 0.014). Unfortunately, treatment response had no independent predictive value for OS and PFS in the multivariate analysis, possibly due to the small sample size of our cohort or the interplay between FMR and treatment response.

**Table 4:** Univariate and multivariate analysis of factors associated with overall survival and progression-free survival

Characteristics	Overall survival			Progression-free survival	
	HR (95% CI)	***p***		HR (95% CI)	***p***
**Univariate analysis**					
Age (< 65 years/≥ 65 years)	4.541 (1.694–12.172)	0.003	2.414 (1.033–5.641)	0.042	
Gender (male/female)	0.841 (0.319–2.219)	0.727	0.689 (0.283–1.681)	0.414	
Location (upper/middle/lower)	0.812 (0.437–1.508)	0.509	0.710 (0.401–1.256)	0.239	
Length (< 5 cm/≥ 5 cm)	1.905 (0.773–4.697)	0.161	1.814 (0.807–4.078)	0.150	
Clinical stage (IIb/III)	2.975 (0.982–9.015)	0.054	2.405 (0.932–6.206)	0.070	
Response (OR/non-OR)	2.442 (0.973–6.126)	0.057	2.587 (1.096–6.109)	0.030	
BF	1.983 (0.811–4.852)	0.134	1.821 (0.787–4.214)	0.162	
BV	1.181 (0.489–2.853)	0.712	1.234 (0.552–2.762)	0.608	
TTP	1.507 (0.631–3.600)	0.335	1.681 (0.725–3.898)	0.226	
SUVmax	1.088 (0.458–2.587)	0.848	1.240 (0.555–2.769)	0.600	
MTV	1.308 (0.554–3.088)	0.540	1.780 (0.761–4.162)	0.183	
TLG	1.308 (0.554–3.088)	0.540	1.780 (0.761–4.162)	0.183	
FMR	3.092 (1.245–7.678)	0.015	2.829 (1.202–6.661)	0.017	
**Multivariate analysis**					
Age(< 65 years/≥ 65 years)	7.446 (2.273–24.395)	0.001	2.592 (1.081–6.213)	0.033	
Clinical stage (IIb/III)	3.790 (1.104–13.013)	0.034	NA	0.245	
Response (OR/non-OR)	NA	0.867	NA	0.427	
FMR	2.914 (1.138–7.462)	0.026	3.009 (1.252–7.227)	0.014	

*OR* objective response, *BF* blood flow, *BV* blood volume, *TTP* time to peak, *SUVmax* maximum standardized uptake value, *MTV* metabolic tumor volume, *TLG* total lesion glycolysis, *FMR* flow-metabolism ratio, *HR* hazard ratio, *95% CI* 95% confidence interval

## Discussion

In the present study, we measured tumor perfusion and metabolism in parallel in patients with LA ESCC who were going to undergo dCRT. Our preliminary results showed that the FMR can serve not only as a predictor of tumor response to therapy but also as an independent prognostic indicator for PFS and OS. The integration of perfusion CT and ^18^F-FDG PET/CT imaging provides a novel biomarker that better reflects the biological behavior of tumors than the individual imaging modalities, allowing us to better stratify patients with different clinical outcomes.

Current advances in molecular and functional imaging have provided a range of noninvasive and accurate tools for mapping core hallmarks at the tumor level, including deregulated angiogenesis and metabolism. ^18^F-FDG PET/CT is a well-known imaging technology that can detect abnormalities in tumor glucose metabolism before changes in anatomical images can be observed, and it has been widely used in the clinical practice of EC [12]. However, in our study, we did not find that the values of the baseline PET-related parameters could predict survival and sensitivity to chemoradiation, which was inconsistent with some previous studies [12–15]. This may be due to our small sample size or to the heterogeneity of the selected population. Although tumor perfusion imaging is less commonly used than ^18^F-FDG PET/CT clinically, there are a range of feasible techniques that can be used to measure tumor perfusion, such as perfusion CT, ^15^O-water PET, and dynamic contrast-enhanced magnetic resonance imaging (DCE-MRI) [23]. In the present study, we selected perfusion CT as the tool to assess tumor vascular physiology in vivo because it is a reliable, reproducible, widely available, and effective method, and many previous studies on solid tumors have demonstrated its advantages [5–11, 20, 21, 24, 25]. In studies of EC, Hayano et al. [10] and Makari et al. [11] also applied perfusion CT, confirming the feasibility of perfusion CT in detecting angiogenesis of tumors in hollow organs. More importantly, they consistently found that BF is an effective predictor of treatment response and survival in EC patients receiving chemoradiation [10, 11]. Our conclusions are partially consistent with the above studies because we also demonstrated that tumors with a higher BF before treatment exhibited a better tumor response to chemoradiation. A possible explanation for this is that well-perfused tumors can better carry cytotoxic drugs inside the tumor and may have higher blood oxygen levels, which are considered an optimal radiosensitizer. However, we did not find that a high BF could predict better survival. This may be due to the double-edged sword effect of angiogenesis, that is to say, the high perfusion state of the tumor only temporarily improves the sensitivity of CRT, but its role in promoting tumor progression and metastasis in the long-term is greater than the former. Therefore, high BF can predict better treatment response but not long-term survival.

Many studies have explored the correlation between tumor blood perfusion and metabolic characteristics, and most have concluded that in many tumor types, including NSCLCs [25–27], head and neck cancers [24], liver tumors [28] and pancreatic cancers [16], tumor perfusion, and metabolic activity are often uncoupled or mismatched, especially in larger tumors. For example, Miles et al. [25] reported that in NSCLC, when the tumor is less than 2.4 cm in diameter, BF and metabolism may increase in parallel, but as the tumor grows, BF tends to decrease, and FDG uptake tends to increase, indicating the uncoupling of BF and glucose metabolism. Ippolito et al. [26] and Calandriello et al. [27] arrived at the same conclusion in NSCLC by using perfusion CT and PET/CT. In animal tumor models, Stewart et al. [28] also found a similar phenomenon by dynamic observation; that is, as the tumor grew, the hepatic BF decreased, and the SUV increased. Moreover, in a study of head and neck tumors, investigators [24] also found an uncoupling trend between perfusion and glucose utilization in larger tumors. Therefore, it is logical to assume that this mismatch between BF and metabolism is common in malignant tumors. In the current research, no correlation was observed between parameters representing tumor perfusion and glucose metabolism, including between BF and SUV, suggesting that this mismatch may also exist in ESCC. Many theories focusing on “hypoxia” have been established to explain this BF-metabolism mismatch that occurs during tumor growth [18, 19, 21]. Compared with normal tissues, the vascular physiology of malignant tumors is often deformed. As the tumor grows, this malformed microvascular system cannot provide sufficient blood supply and oxygen for the proliferation of tumor cells, leading to low oxygen levels in the tumor microenvironment, i.e., hypoxia. However, tumors can adapt to hypoxia by stimulating hypoxia-inducing factor (HIF) expression, which can accelerate anaerobic glycolysis by increasing the expression of Glu-1 glucose transporters and hexokinase, ultimately helping tumor cells survive the dilemma [29]. Therefore, the mismatch of high metabolism and low blood vascularity in tumors indicates this adaptive response to hypoxia [18, 21].

The adaptive response of the tumor to hypoxia may indicate that the tumor has an aggressive biological phenotype and greater therapeutic resistance. Indeed, a number of studies have demonstrated that the flow-metabolism mismatch is closely related to poorer therapy response and long-term prognosis [16–19, 22]. In a study of pancreatic tumors, Komar et al. [16] reported that malignant lesions exhibited decreased BF and increased SUVmax compared to normal pancreatic tissue. Moreover, they found that a high ratio of SUVmax to BF was a strong predictor of poor survival. In another study of pancreatic tumors, Chen et al. investigated the prognostic significance of imaging biomarkers derived from multiparametric PET/MRI. They found that the TLG/*peak* ratio was not only able to predict OS but also outperformed other parameters. Although different from Komar’s study, Chen used PET-derived TLG and MRI-derived *peaks* instead of SUV and BF to quantify tumor metabolism and angiogenesis, and the conclusions were consistent, confirming that the flow-metabolism mismatch was correlated with poorer survival [22]. In breast cancer, researchers [17] concluded that patients with higher glucose metabolism-to-BF ratios not only had shorter disease-free survival but also exhibited a worse clinical response to neoadjuvant chemotherapy. They believe that this result is related to hypoxia-induced chemotherapy resistance, which reduces treatment effectiveness. Miles and Williams comprehensively reviewed the mismatch between BF and tumor metabolism, showing that it exists in a variety of solid tumors and is associated with poor biological behavior [18]. Our conclusions are consistent with those of Komar et al. [16] or Mankoff et al. [17]. To the best of our knowledge, this is the first prospective study to propose the FMR as a promising imaging biomarker to predict the chemoradiation sensitivity and prognosis of ESCC. More specifically, we found that patients with a low FMR responded poorly to chemoradiation and had shorter PFS and OS than patients with a high FMR. The findings of Goh et al. and Wang et al. may explain why a lower FMR is associated with poor clinical outcomes [30, 31]. Goh et al. [30] observed that the low-flow and high-metabolism phenotype (i.e., a lower FMR) was associated with higher HIF-1 expression in primary colorectal cancer, indicating tumor adaption to hypoxia. Wang et al. [31] confirmed that FMR was significantly and inversely related to hypoxic parameters (measured by partial pressure of oxygen or imidazole staining) and, more specifically, that hypoxia was more pronounced in tumor regions with low perfusion and high metabolism. Hypoxia, in turn, can induce the proliferation of more aggressive cancer cells and resistance to systemic treatment [32]. Therefore, hypoxia-mediated cancer aggressive behavior may explain poorer patient outcomes predicted by a low FMR.

Although the combined use of functional imaging is currently not very common, it is definitely a trend that can help us provide multifaceted and unique information about tumor characteristics and aggressiveness, thereby aiding in the development of individualized treatment plans. The results of our pilot study fully prove this point. For patients with a low FMR, it may be necessary to increase the intensity of chemotherapeutic drugs and the dose of radiation therapy or combine them with other therapies to control tumors and thus improve survival. In addition, multiparameter functional imaging may also provide valuable indicators for screening patients who may benefit from antiangiogenic therapy or immunotherapy, which will be the focus of future research.

However, we must admit that the current research has some limitations. First, this is a small sample study with patients from a single center. Second, although we have used endoscopic ultrasonography, enhanced CT, and PET/CT to determine the clinical TNM stage, it may still not be as accurate as the pathological TNM stage, especially the N stage, because some metastatic lymph nodes are relatively small, and PET cannot recognize them. Third, we only focused on the impact of baseline perfusion and metabolic parameters on the therapy response and long-term survival of patients. In future research, we will dynamically monitor the changes in related parameters during chemoradiation and may find more valuable biomarkers. Finally, the threshold values of the FMR for predicting chemoradiotherapy sensitivity and survival were not determined, and further research is needed to clarify these values.

## Conclusions

The flow-metabolism mismatch demonstrated by a low FMR shows good potential in predicting chemoradiotherapy sensitivity and survival in ESCC. Therefore, as a promising biomarker, FMR may be helpful in the selection of personalized treatment, thus changing the management and improve the prognosis for ESCC patients.

## Data Availability

The datasets used and/or analyzed during the current study are available from the corresponding author on reasonable request.

## Abbreviations

LA ESCC: Locally advanced esophageal squamous cell carcinoma
dCRT: Definitive chemoradiotherapy
TNM: Tumor, lymph node, metastasis
DCE: Dynamic contrast-enhanced
CT: Computed tomography
^18^F-FDG PET: ^18^F-fluoroglucose glucose positron emission tomography
FMR: Flow-metabolism ratio
BF: Blood flow
BV: Blood volume
TTP: Time to peak
SUVmax: Maximum standardized uptake value
MTV: Metabolic tumor volume
TLG: Total lesion glycolysis
RECIST: Revised Response Evaluation Criteria in Solid Tumors
CR: Complete response
PR: Partial response
SD: Stable disease
PD: Progressive disease
ROC: Receiver operating characteristic
OS: Overall survival
PFS: Progression-free survival
AUC: Area under the ROC curve
ORR: Objective response rate
HIF: Hypoxia-inducing factor
